# A Case of Nasopharyngeal Tuberculosis with Cervical Lymph Node Tuberculosis Suspected of Cervical Malignant Disease at the First Examination

**DOI:** 10.3390/clinpract11010008

**Published:** 2021-01-29

**Authors:** Takeshi Kusunoki, Hirotomo Homma, Yoshinobu Kidokoro, Akihisa Yoshikawa, Kumiko Tanaka, Satoko Kubo, Ryo Wada, Katsuhisa Ikeda

**Affiliations:** 1 Department of Otorhinolaryngology, Juntendo University of Medicine, Shizuoka Hospital, Shizuoka 410-2295, Japan; h-honma@juntendo.ac.jp (H.H.); ykidoko@juntendo.ac.jp (Y.K.); a-yoshikawa@juntendo.ac.jp (A.Y.); k-tanaka@juntendo.ac.jp (K.T.); s-kubo@juntendo.ac.jp (S.K.); 2 Departement of Pathology, Juntendo University of Medicine, Shizuoka Hospital, Shizuoka 410-2295, Japan; mdksmrwjunten522@ybb.ne.jp; 3 Department of Otorhinolaryngology, Faculty of Medicine, Juntendo University of Medicine, Tokyo 113-0034, Japan; ike@juntendo.ac.jp

**Keywords:** nasopharyngeal tuberculosis, cervical lymph node tuberculosis PET-CT

## Abstract

A case of nasopharyngeal tuberculosis with cervical lymph node tuberculosis is reported. The patient was a 20-year-old female immigrant from Vietnam and cook apprentice. Her chief complaint was left neck swelling with pain for three months. She was diagnosed with left neck lymphadenitis at a previous hospital, which suspected malignant lymphoma and referred her to our hospital. At the time of the first visit, she had left lymph swelling with tenderness and granuloma-like masses in the nasopharynx. PET-CT showed accumulations in both the swollen left neck lymph and nasopharynx. The diagnosis of this case would appear to be nasopharyngeal cancer with left and neck lymph node metastasis or nasopharyngeal tuberculosis with cervical lymph node tuberculosis in addition to malignant lymphoma. Based on some examinations (biopsy, bacteria culture, and imaging), it was diagnosed as nasopharyngeal tuberculosis with cervical lymph node tuberculosis. Therefore, she was treated with anti-tuberculosis agent in respiratory medicine.

## 1. Introduction

In Japan, the most common in extrapulmonary tuberculosis has been tuberculosis pleurisy, followed by lymph node tuberculosis. In particular, cervical lymph node tuberculosis is accounts for 60% in lymph node tuberculosis [1]. Therefore, the otolaryngologist could encounter it and would require differentiation with head and neck malignant diseases [2]. We experienced a case of upper pharyngeal tuberculosis with cervical lymph node tuberculosis. At the first examination, this case diagnosis was necessary to be distinguished form nasopharyngeal cancer with left or malignant lymphoma. This case report presents the clinical imaging, bacterial, histopathologic examination and some considerations.

## 2. Case Report

Patient: 20-years -old, female, Vietnamese

Occupation: Culinarian Apprentice

Chief complaint: Left neck swelling

**Figure 1:**
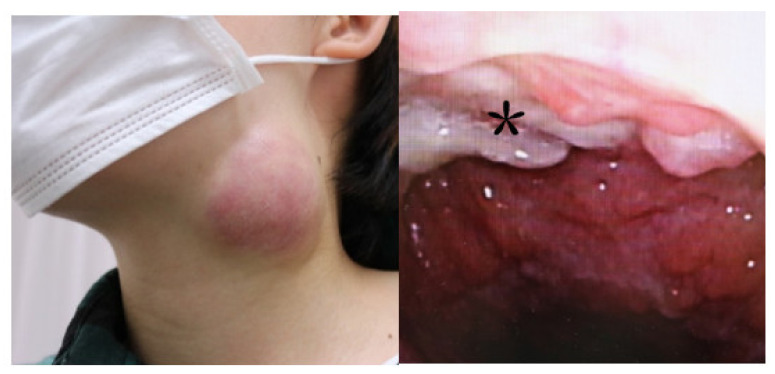
At first examination, the patient showed left submandibular lymph swelling (**left**) and granuloma-like masses (an asterisk) in the nasopharynx (**right**).

**Figure 2:**
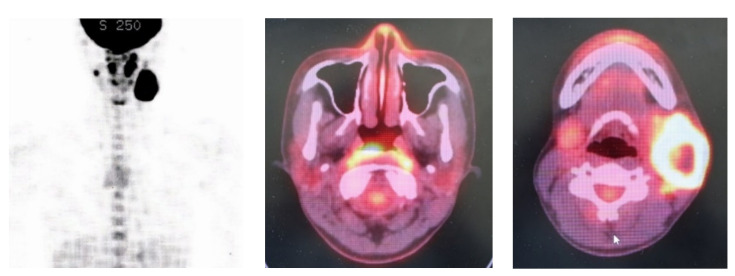
PET-CT could show accumulations in both the left neck lymph and nasopharynx, but nowhere else in the body.

Her chief complaint was left neck swelling with pain three months before. She was diagnosed as left neck lymphadenitis by the hospital of the referral and administrated antibiotics, but did not improve. Therefore, this hospital suspected malignant lymphoma and introduced her to our hospital. At the first examination, she had left lymph swelling with tenderness and granuloma-like masses in the nasopharynx (Figure 1). PET-CT showed accumulations in both the left neck lymph and nasopharynx, but not in other parts of the body (Figure 2). Chest CT and X-rays showed no abnormal findings in the lung. Both of her ear drums and middle ears appeared normal by otoscope and CT. Concerning her diagnosis, we considered the possibility of nasopharyngeal cancer with left and neck lymph node metastasis or nasopharyngeal tuberculosis with cervical lymph node tuberculosis, in addition to malignant lymphoma.

For the differential diagnosis, she underwent a biopsy of the nasopharyngeal masses, tuberculosis culture, polymerase chain reaction (PCR) by fine needle puncture suction and fine needle aspiration (FNA) cytology for the left lymph node. Additionally, tuberculosis culture and PCR from the sputum and gastric juice were performed.

**Figure 3:**
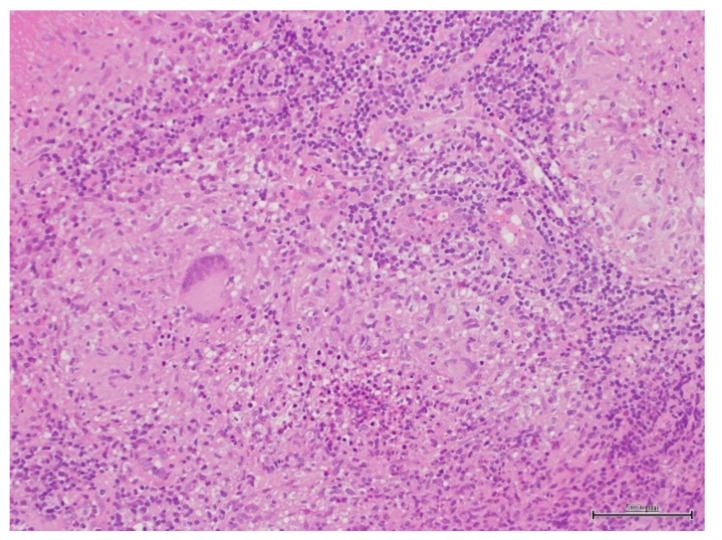
Histopathologic findings of nasopharyngeal biopsy showed granuloma. With epithlioid cells and giant cells with necrotic lesions (hematoxylin-eosin staining).

Biopsy results of the nasopharyngeal masses revealed epithelial cell granuloma with necrosis, which are characteristic findings of tuberculosis (Figure 3). The finding of the FNA of the left neck lymph node was benign lymph nodes with no malignant cells.

Sputum, gastric juice and lymph puncture solution were negative in the Ziehl-Neelsen staining and not detected in the culture. However, the tuberculosis PCR of the left neck lymph node puncture liquid was positive, but negative in the tuberculosis culture of the left neck lymph node puncture solution. Tuberculosis culture and PCR of both sputum and gastric juice were negative. From the above examination results, it was diagnosed as nasopharyngeal tuberculosis with cervical lymph node tuberculosis. Therefore, she was treated with an anti-tuberculosis agent by the respiratory medicine department. At present, 9 months have passed since the start of treatment. The upper pharynx lesion and cervical lymph node swelling disappeared in the first 3 months of anti-tuberculosis administration.

## 3. Discussion

In this case of nasopharyngeal tuberculosis together with cervical lymph node tuberculosis, the clinical findings showed a nasopharyngeal mass and left neck lymphadenopathy. This patient had no nasal symptoms, headache or ophthalmological findings. When cervical lymph node swelling is observed, it is necessary to consider the possibility of cervical lymph node metastasis from head and neck cancer, except for infections or lymphoma. As one of the examinations, nasopharyngeal endoscopy was performed, which revealed a nasopharyngeal mass. PET-CT also showed accumulation at the same site. PET-CT can determine the spread of tuberculous lesions as well as tumors and lymphomas. Champion [3] reported that PET-CT would be a useful tool for the diagnosis and evaluation of the nasopharyngeal tuberculosis.

Basal et al. [2] described that nasopaharyngeal tuberculosis could appear clinically similar to nasopaharyngeal carcinoma. Therefore, in order to distinguish tuberculosis from head and neck malignant diseases, Darouassi et al. [4] recommend biopsy and bacteriological examination. Our biopsy of nasopharyngeal masses revealed epithelial cell granuloma with necrosis, which are characteristic findings of tuberculosis. FNA of the left neck lymph node, malignant cells were not found. Tuberculosis bacteria were not detected in the sputum, gastric juice or lymph puncture solution in culture. This could mean that the tubercle bacilli had already died, were reduced in number or decreased in the ability to grow. However, tuberculosis in the PCR of the left neck lymph node puncture liquid was positive, confirming the diagnosis of nasopharyngeal tuberculosis with cervical lymph node tuberculosis. We did not examine for the presence of HIV or other viruses or bacteria. However, this patient had no immunodeficiency symptoms and the characteristic pathological findings of tuberculosis were recognized by the upper pharynx biopsy. Moreover, the upper pharyngeal lesion and cervical lymph node swelling disappeared in the first 3 months of anti-tuberculosis administration. This suggests that tuberculosis infection was the main etiology of this case.

In our case, PET-CT revealed accumulations in both the left neck lymph and nasopharynx, but not in other parts of the body. Chest CT and X-rays showed no abnormal findings in the lung. Mahindra et al. [5] reported that fewer than 20 percent of patients with nasopharyngeal tuberculosis have an abnormality in the lung. Some researchers [2,4,6,7] have presented cases of nasopharyngeal tuberculosis with cervical lymph node tuberculosis as in our case. Therefore, cases of cervical lymph node tuberculosis should be checked for nasopharyngeal tuberculosis as well as lung tuberculosis.

Our case showed no otitis media. Nasopharyngeal tuberculosis might be associated with tuberculous otitis media via the eustachian tube from the nasopharynx. Since Sugimoto et al. [7] observed tuberculosis otitis in 7 out of 19 cases of nasopharyngeal tuberculosis in Japan, cases of nasopharyngeal tuberculosis should also be examined for otitis media, as well. Conversely, cases of tuberculosis otitis media should be checked for the presence of nasopharyngeal tuberculosis

## Data Availability

The data of this article are not publicly available to protect personally identifiable information. And are available for requests of the corresponding authors.
